# Novel siRNA Delivery System to Target Podocytes *In Vivo*

**DOI:** 10.1371/journal.pone.0009463

**Published:** 2010-03-01

**Authors:** Peter V. Hauser, Jeffrey W. Pippin, Cora Kaiser, Ronald D. Krofft, Paul T. Brinkkoetter, Kelly L. Hudkins, Dontscho Kerjaschki, Jochen Reiser, Charles E. Alpers, Stuart J. Shankland

**Affiliations:** 1 Division of Nephrology, University of Washington, Seattle, Washington, United States of America; 2 Department of Pathology, University of Washington, Seattle, Washington, United States of America; 3 Department of Clinical Pathology, Vienna Medical University, Vienna, Austria; 4 Division of Nephrology & Hypertension, University of Miami, Miami, Florida, United States of America; L' Istituto di Biomedicina ed Immunologia Molecolare, Consiglio Nazionale delle Ricerche, Italy

## Abstract

Podocytes are injured in several glomerular diseases. To alter gene expression specifically in podocytes *in vivo*, we took advantage of their active endocytotic machinery and developed a method for the targeted delivery of small interfering ribonucleic acids (siRNA). We generated an anti-mouse podocyte antibody that binds to rat and mouse podocytes *in vivo*. The polyclonal IgG antibody was cleaved into monovalent fragments, while preserving the antigen recognition sites. One Neutravidin molecule was linked to each monovalent IgG via the available sulfohydryl group. Protamine, a polycationic nuclear protein and universal adaptor for anionic siRNA, was linked to the neutravidin via biotin. The delivery system was named *shamporter* (***s*** **h**eep ***a***nti ***m***ouse ***po***docyte trans***porter***). Injection of *shamporter* coupled with either nephrin siRNA or TRPC6 siRNA via tail vein into normal rats substantially reduced the protein levels of nephrin or TRPC6 respectively, measured by western blot analysis and immunostaining. The effect was target specific because other podocyte-specific genes remained unchanged. *Shamporter +* nephrin siRNA induced transient proteinuria in rats. Control rats injected with *shamporter* coupled to control-siRNA showed no changes. These results show for the first time that siRNA can be delivered efficiently and specifically to podocytes *in vivo* using an antibody-delivery system.

## Introduction

Podocytes are highly specialized terminally differentiated epithelial cells. Together with the glomerular basement membrane (GBM) and endothelial cells, they comprise the glomerular filtration barrier of the kidney. Podocytes are injured in a variety of acquired and congenital diseases by immune and non-immune mediated mechanisms [1], [2]. Although genetic approaches have made enormous contributions to our understanding of podocyte disease, the generation of animals by specific gene engineering are typically limited to mice [3], although several groups have recently reported on gene manipulation in rats [4]. The genetic approach however, is time consuming and costly, and cannot be performed in man. Moreover, in contrast to several well defined and characterized experimental podocyte disease models in rats such as the passive Heymann nephritis, puromycin nephrosis, the remnant kidney model and others [5], the number of mouse models available to study podocyte diseases are limited in number, and are also substantially less well defined.

Thus, from an investigational and potentially therapeutic standpoint, the ability to modify genes in established podocyte disease models as well as to have the ability to alter expression in man is desirable.

In order to address this goal, we employed the use of RNA interference (RNAi) [6], [7]. RNAi has advantages in that it can reduce the expression of genes that are either constitutively expressed in cells, or genes that are increased following a stimulus such as injury. The commonly used methods used to transfer RNAi molecules into cells in culture include electroporation, lipid-based transfection reagents or nanoparticles. Unfortunately, when employed *in vivo*, these methodologies are not cell specific, thus limiting their utility *in vivo* for delivering RNAi to target specific organs or specific cell types within that organ. Recent evidence has emerged that podocytes have a robust machinery for endocytosis [8], which may be statin dependent [9]. It has also been shown that podocytes utilize an IgG and albumin transport mechanism to remove IgG from the glomerular basement membrane (GBM) [10]. In this study, we took advantage of podocyte endocytosis to devise a novel method for podocyte specific uptake of siRNA *in vivo*, allowing specific modulation of gene expression.

## Results

### Construction of the Delivery Antibody

**Figure 1:**
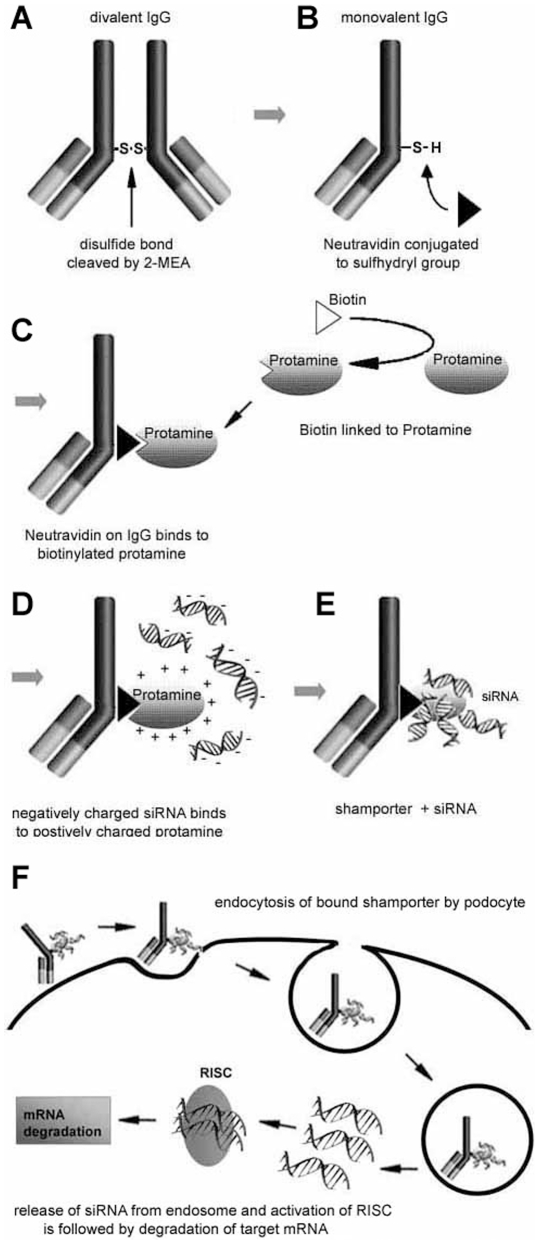
Design of *Shamporter* (sheep anti mouse podocyte & transporter). *Shamporter* is a modified anti podocyte antibody that piggybacks siRNA to a target cell. (**A**) Specific cleaving of the anti mouse podocyte divalent IgG at the inter-heavy chain disulfide bond, using 2-Mercaptoethylamine, results in monovalent IgG. (**B**) A Neutravidin binding site is conjugated to the available sulfohydryl group. (**C**) Protamine is biotinylated which then binds to the monovalent IgG. (**D** and **E**) Negatively charged siRNA molecules bind to the positively charged protamine domain of the *shamporter* construct. (**F**) The modified antibody (*shamporter*) carrying siRNA binds to the podocytes. The bound antibody is internalized and transported to the cytoplasm. Uncoating of the endosomatic vesicle releases the transported siRNA into the cytoplasm. The released siRNA activates RISC (RNA induced silencing complex), which is followed by degradation of the target mRNA.

Central to this technique is an anti mouse podocyte antibody generated in sheep. The antibody was modified so that (i) it could be used as a vehicle to deliver small nucleic acids (siRNA) to podocytes *in vivo* and (ii) to minimize stimulation of the host immune system, such as complement activation. Modification and the hypothetical mode of action of the antibody is shown in **Figure 1** and described in the method section.

### Affinity of the Anti-Podocyte Antibody

**Figure 2:**
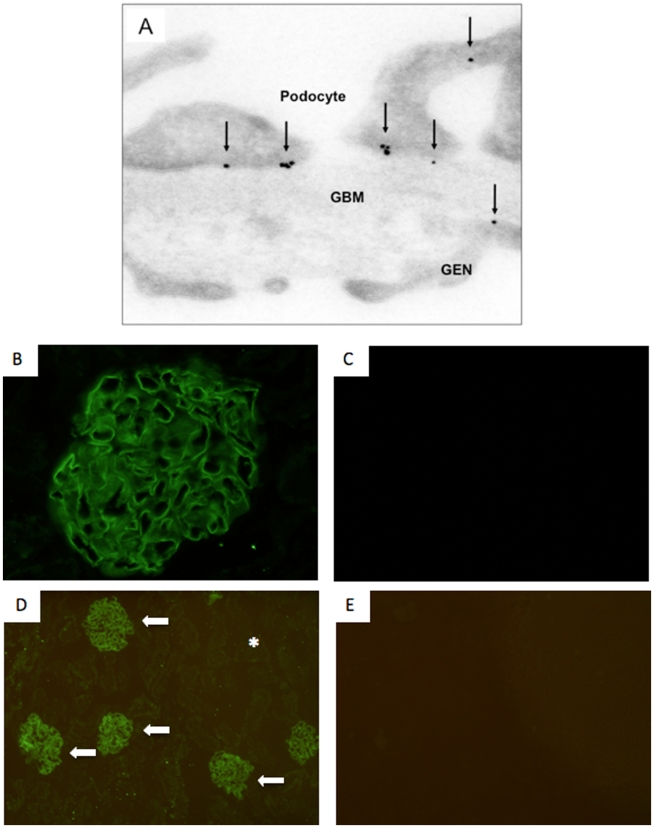
Affinity of the anti-podocyte antibody. The antibody binds to the apical, basal and the slit diaphragm regions of the podocyte (**A**). A small number of immunogold particles were present in the glomerular basement membrane (GBM) and endothelial cells (GEN). The arrows indicate examples of immunogold particles. Sheep anti mouse podocyte antibody staining was detected only in the glomerulus (**B + D arrows**) and was absent in the tubular compartment (**D asterix**). Control animals injected with normal sheep IgG showed no staining (**C + E**).

Immunogold staining of normal mouse kidneys was performed to determine the cellular affinity of the antibody generated for these studies. Our results revealed the presence of immunogold particles in the glomerulus, predominantly in podocytes (**Figure 2.A**). A very small number of immunogold particles also bound endothelial cells in mouse glomeruli (**Figure 2.A**). These results show that the antibody used in these studies bound predominantly to antigen(s) on podocytes. To test for potential uptake of the podocyte antibody by the proximal tubular cells, antibody was injected into rats. Immunofluorescent staining for sheep anti podocyte IgG was detected only in the glomerulus (**Figure 2.B**). No staining was observed in the tubulointerstitial region, consistent with the notion that uptake of the antibody by tubular cells was absent, and is thus glomerular limited (**Figure 2.D**).

### Anti-Podocyte Antibody Localizes to Podocyte Membranes

**Figure 3:**
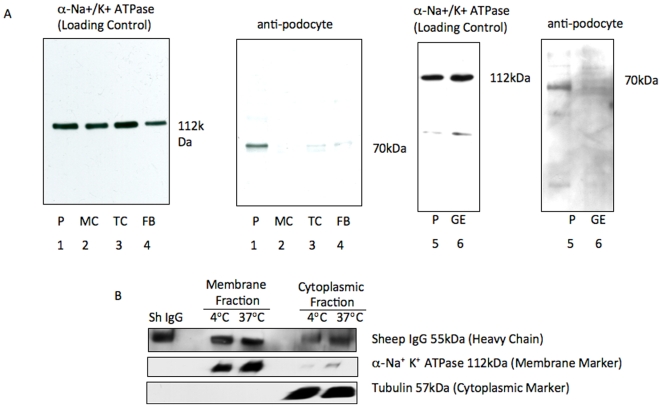
Podocyte antibody specificity and uptakes by podocytes. Western blot analysis with the podocyte antibody on membrane fractions from cultured immortalized mouse podocytes (P), mouse mesangial cells (MC), mouse fibroblasts (FB), rat proximal tubular epithelial cells (TC), and rat glomerular endothelial cells (GE), showed a band only in podocytes (**A lanes 1 & 5**). After 30 minutes at 37°C the cytoplasmic fraction of podocytes incubated with *shamporter*, showed a marked increase in detectable sheep IgG (55 kDa). Incubation of cells on 4°C instead significantly slowed *shamporter* uptake (**B**).

To validate that the anti-podocyte antibody indeed binds to the cell membrane of podocytes and not other glomerular cells, membrane fractions of cultured immortalized mouse podocytes (P), mouse mesangial cells (MC) [11], mouse fibroblasts (FB) [12], rat proximal tubular epithelial cells (TC) [13], and rat glomerular endothelial cells (GE) [14] were analyzed by western blot analysis. The results of immunoblotting with anti-podocyte antibody are shown in **Figure 3**. A dominant band at a molecular weight of 70 kD was observed in the membrane-rich fraction from podocytes (**Figure 3.A Lane 1 & 5** ). The 70 Kd band however was not detected in the membrane fractions from MC, FB, TC, or GE (**Figure 3.A, Lanes 2–4 & 6**). Sodium potassium adenosine-triphosphate (Na+ K+ ATPase) was used as loading control for membrane protein.

### Organ Specificity of Podocyte Antibody *In Vivo*

To ensure that the sheep anti-podocyte antibody did not bind to cells in other organs beyond the glomerulus, male Balb-c mice were injected with anti-podocyte antibody and several organs harvested 72 hours later (**Figure S1**). Within the kidney, staining was detected only in glomeruli, and this was in a podocyte distribution. In contrast, IgG staining was not detected in the lungs, liver, muscle or colon (not shown), similar to control mice injected with normal sheep IgG. As expected, positive staining for both the anti podocyte antibody and normal sheep IgG used as control was detected in the spleen. These results are consistent with non-specific binding or trapping of IgG in the spleen (**Figure S1.C** and **S1.D**). Faint positive staining for sheep anti-mouse podocyte IgG was also detected in the brain vasculature, but not in the grey matter or neuronal cells (**Figure S1.E** and **S1.F**). No other vasculature stained positive. Affinity testing of the anti-podocyte antibody was performed using the original unmodified antibody, since cleaving of the internal heavy chain disulfide bond alters the Fc-portion of the antibody [15], [16].

### *Shamporter* Uptake by Podocytes

Subcellular protein fractioning, followed by western blot analysis for sheep IgG, was used to detect anti podocyte antibody uptake by cultured mouse podocytes. Sheep anti-podocyte antibody was applied to cultured immortalized mouse podocytes for 30 minutes on ice, and then unbound IgG was washed away. Western blot for sheep IgG heavy chain (55 kDa band) showed that sheep IgG readily bound to the membrane fraction of podocytes (**Figure 3**). Following additional 30 minute incubation at 37°C a strong band for sheep IgG heavy chain was detected in the cytoplasmic fraction. Alpha-Tubulin (cytoplasmic) and Na+ K+ ATPase (membrane) were used as loading controls. Taken together, these results show active IgG internalization by podocytes.

### *Shamporter* Delivery of siRNA Reduces Protein Levels *In Vitro*

**Figure 4:**
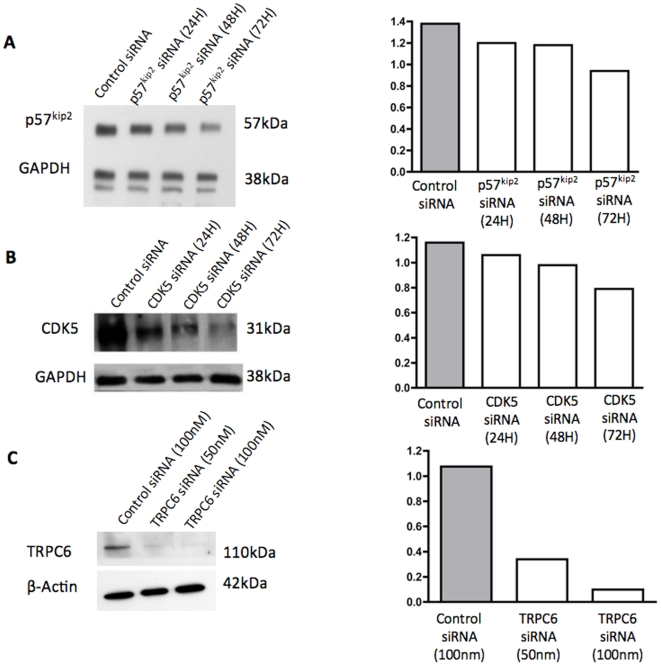
Western blot analyses for p57, CDK5 and TRPC6. In cultured immortalized mouse podocytes transfected with *shamporter* + siRNA directed against p57 (**A**) or CDK5 (**B**), there was a progressive decrease in protein levels at 48 h and 72 hours. In cultured podocytes transfected with *shamporter* + siRNA directed against TRPC6, there was a dose dependent decrease in protein levels for TRPC6 (**C**). Cells transfected with nonfunctional control-siRNA served as control. The panels on the right show densitometry performed against loading controls GAPDH or β-Actin.

To ensure that our construct was efficient at transferring siRNA into podocytes and knocking down target genes, the delivery system was tested *in vitro*. Therefore, siRNA specific to the cyclin dependent kinase inhibitor p57Kip2, cyclin dependent kinase 5 (CDK5) and transient receptor potential channel 6 (TRPC6), which are constitutively expressed in podocytes [17]–[19], were coupled to the *shamporter* construct and administered to differentiated immortalized mouse podocytes in culture. The results show that compared to control siRNA, *shamporter* + p57Kip2 siRNA reduced p57Kip2 protein to 87% of control after 24 hours, 85% after 48 hours and 68% of control after 72 hours (**Figure 4.A**). Exposing podocytes to *Shamporter* + CDK5 siRNA reduced CDK5 protein levels to 92% of control after 24 hours, 84% after 48 hours and 68% after 72 hours compared to control cells exposed to control siRNA (**Figure 4.B**). Exposing podocytes to *shamporter* + TRPC6 siRNA reduced TRPC6 levels in a dose dependent manner (**Figure 4.C**). These results demonstrate that the *shamporter* delivery system is effective at getting into podocytes and specifically decreasing the protein levels of target genes in cultured podocytes.

### *Shamporter* Delivery of Nephrin and TRPC6 siRNA Reduces Protein Levels *In Vivo*

**Figure 5:**
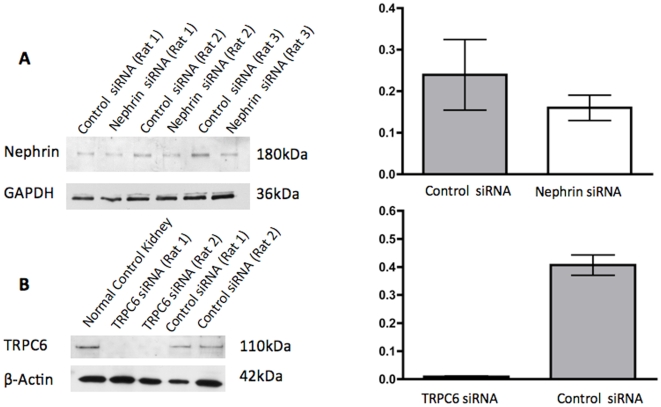
Western blot analysis for Nephrin and TRPC6 in rats injected with *shamporter* and siRNA. Rats were injected via tail vein with *shamporter* + control siRNA or *shamporter* + nephrin siRNA or *shamporter* + TRPC6 siRNA. Compared with control siRNA injected rats, there was a significant decrease in nephrin protein levels in the rats injected with nephrin siRNA (**A**). Likewise, there was a significant decrease in TRPC6 protein levels in the rats injected with TRPC6 siRNA (**B**). Staining for GAPDH or beta actin was used as a protein loading control, and the podocyte specific protein podocin or nephrin were used to ensure that the same amount of podocyte protein was loaded. Densitometry and of the results are shown in the graph. Ratios were compared using t-test (p<0.05); ratios are expressed as median ± min and max values.

To test delivery of siRNA coupled to *shamporter in vivo*, siRNA directed against nephrin or TRPC6 coupled to *shamporter* was injected intravenously into uninephrectomized rats (**Figure 5**). Western blot and densitometry in **Figure 5.A** demonstrates reduced glomerular expression of nephrin in rats injected with *shamporter* + nephrin siRNA. Nephrin levels are expressed in ratio to glyceraldehyde-3-phosphate dehydrogenase (GAPDH) used as a loading control. To further test the reproducibility of *shamporter* targeted delivery *in vivo*, siRNA directed against another podocyte protein, (TRPC6) was injected. Western blot analysis and densitometry for TRPC6 on glomerular protein from rats injected with *shamporter* coupled to TRPC6 siRNA shows a substantial reduction in TRPC6 levels compared with rats injected with *shamporter* coupled negative control siRNA (**Figure 5.B**).

**Figure 6:**
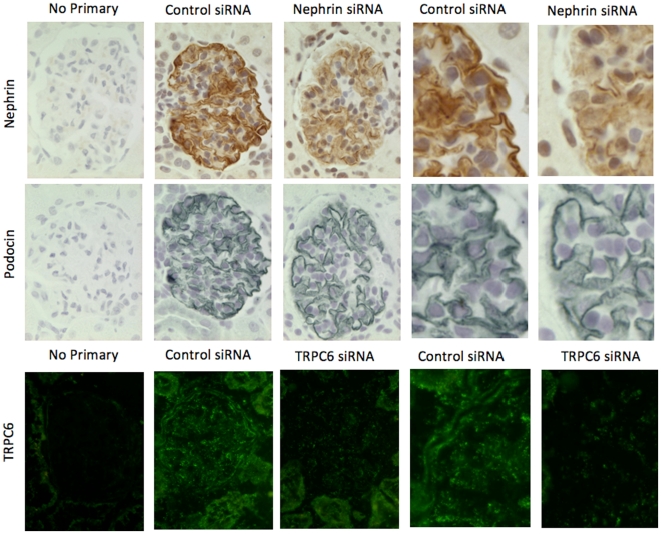
Immunohistochemistry for nephrin, podocin and TRPC6. Animals injected with *shamporter* + control siRNA show a strong linear staining signal for nephrin and podocin, as well as glomerular staining for TRPC6. In animals injected with *shamporter* + nephrin or TRPC6 specific siRNA, the staining signal was significantly reduced, while the signal for podocin remained unchanged. When nephrin, podocin, or TRPC6 antibodies were omitted, no staining was detected.

Immunohistochemistry on serial kidney sections of rats was used as another method to demonstrate reduced nephrin levels in animals injected with *shamporter +* nephrin siRNA. As shown in **Figure 6**, rats injected with *shamporter +* nephrin siRNA exhibit a marked decrease in nephrin specific staining, compared with animals injected with control siRNA. In contrast, there was no difference in staining for podocin, used as a control. Likewise, immunofluorescent staining was performed on rats injected with *shamporter +* TRPC6 siRNA. There was also a decrease in TRPC6 staining when compared with animals injected with control siRNA. Interestingly, the staining in the glomerular endothelium remained. Taken together, these results show that coupling siRNA to *shamporter* was effective in reducing nephrin and TRPC6 levels *in vivo* in normal rats.

### *Shamporter* Does Not Activate Complement

The potential activation of complement by *shamporter* was ruled out by immunofluorescent staining for rat C5b-9 and C3 on tissue from *shamporter* + control and *shamporter +* nephrin siRNA injected rats (**Figure S2**). Rats with Passive Heymann nephritis (PHN), which are characterized by complement activation, served as positive controls for the C5b-9 and C3 immunostaining.

### Nephrin siRNA Induces Proteinuria

**Figure 7:**
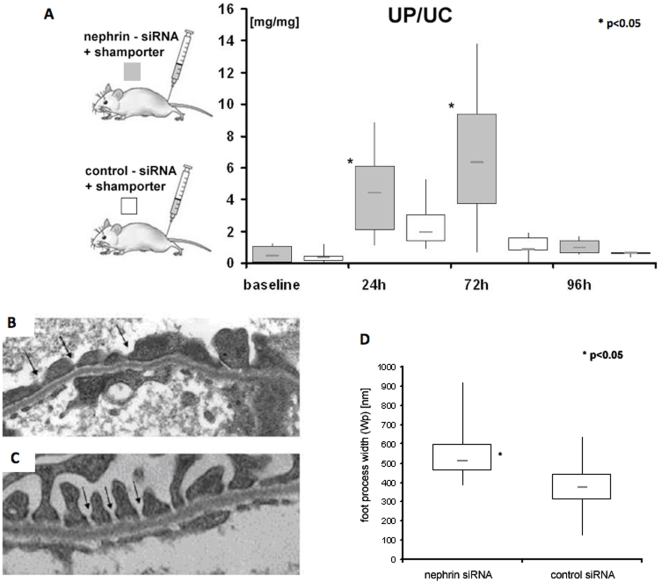
Proteinuria and Podocyte abnormalities induced in nephrin siRNA injected rats. Urinary protein (UP) to urinary creatinine (UC) ratio (mg/mg) was used to assess renal function. UP∶UC ratio was increased significantly in rats injected with *shamporter* + nephrin siRNA after 24 and 72 h compared to baseline (ANOVA; p<0.001). 72 h after injection, *shamporter* + nephrin siRNA injected rats exhibited a significantly higher UP∶UC ratio then animals from the control group (t-test; p<0.005). No significant differences in UP∶UC ratio was found between control animals at 72 h and baseline (**A**) No difference was found in BUN in either group (data not shown). There were no abnormalities in podocytes in control rats injected with *shamporter* + control siRNA (**C**). Arrows indicate normal slit diaphragms. In rats injected with *shamporter* + nephrin siRNA, there was loss of normal slit diaphragm architecture which included crowding of the slit diaphragms and reduced space between adjacent foot processes (**B arrows**). Quantification of foot process width showed a significant increase in rats injected with *shamporter* + nephrin siRNA compared with rats injected with *shamporter* + control siRNA (**D**).

To assess the biological effect of reducing nephrin levels we measured the urine protein to creatinine ratios (UP∶UC) 24 and 72 h after *shamporter* + nephrin siRNA injection. A significant increase (ANOVA; p<0.001) in proteinuria was detected in rats injected with *Shamporter* + nephrin siRNA at 24 and 72 hours compared with baseline (**Figure 7**). There was a slight, but insignificant increase in proteinuria in control animals at 24 h, which returned to baseline by 72 h. The transient increase in the UP∶UC ratio at 24 h in the control group can be explained by the excretion of the normal sheep IgG which was used as a carrier protein. A significant difference was found between experimental group and control at 72 h (t-test; p<0.05), UP∶UC ratios of both groups normalized to baseline after 96 h. There were no differences in BUN measurements 72 hours after treatment with *shamporter* + either siRNA (data not shown). These data show that reducing nephrin levels with a single injection of *shamporter*+nephrin siRNA induced transient proteinuria.

### Nephrin siRNA Induces Podocyte Effacement

In order to determine if *Shamporter* + nephrin siRNA had any effects on podocytes at the ultrastructural level, electron microscopy was performed on biopsies at 72 hours and the results are shown in **Figure 7**. Injecting *shamporter* + control siRNA had no effect on podocyte morphology (**Figure 7.C**). In contrast, administering *shamporter* + nephrin siRNA caused moderate effacement of foot processes (**Figure 7.B**). There were no changes in the glomerular basement membrane or the glomerular endothelial cells.

To quantitiate these changes, the average podocyte foot process width (Wp) was measured. The data showed that animals treated with *shamporter* + nephrin-siRNA had significantly (p<0.000058) larger (566±157 nm) foot process width compared to control animals treated with *shamporter* + control-siRNA (365±126 nm) (**Figure 6.D**).

## Discussion

Although several therapeutic agents are currently available to treat proteinuria caused by podocyte diseases, none are cell-type specific. As a result, side effects occur due to the delivery of these agents to organs beyond the intended target. Methods to deliver therapeutics or experimental drugs can broadly be divided into methods for systemic delivery and organ or tissue specific. Extensive work has been undertaken to deliver genes to the kidney, most recently very successful using podocyte-specific promoter constructs [20], [21], [22]. However, hydrodynamic gene delivery via tail vein requires large volumes and high speed injection that is not readily achievable in humans. In this manuscript we present a novel strategy to deliver siRNA to podocytes to modulate specific gene expression by packaging siRNA on an antibody that binds podocyte membranes and is internalized by endocytosis.

The rationale for the study design was based in part on the recent observation that podocytes have an active endocytic machinery [8], [9]. Podocytes use a receptor mediated uptake mechanism to internalize IgG. Akilesh S *et al*. showed that podocytes express FcRn, an IgG and albumin transport receptor; mice deficient for FcRn accumulated IgG in the GBM as they age [10]. We therefore reasoned that the binding of antibody to the surface of podocytes would favour internalization of the antibody. Moreover, if the antibody could be used as a delivery system, one could then specifically deliver a drug or other substance such as siRNA to podocytes. The immunogold and immunofluorescent staining, as well as western blot analysis presented in this manuscript showed that the antibody we generated did indeed bind predominantly to podocytes.

The major findings in this study were *shamporter* efficiently piggybacks siRNA into podocytes both *in vitro* and *in vivo* as evidence by a decrease in protein levels and immunostaining. The decrease in nephrin levels by siRNA lead to proteinuria and increased foot process width was consistent with other studies, where a mutation (and hence function) in one or more amino acids of nephrin were shown [23], [24]. Moreover, administering an antibody specific to nephrin also induces proteinuria [25]. We were able to confirm the utility of this delivery system by targeting and decreasing levels of a second slit diaphragm protein found in the podocyte, transient receptor potential channel 6 (TRPC6) [19].

Because protamine sulfate is used to induce transient foot process effacement and proteinuria, a particular concern would be that the protamine used as a nucleic acid carrier in the *shamporter* construct might cause changes in the podocyte itself. The mechanism by which protamine induces changes in the podocyte is its cationic charge. However, studies have shown that exposing protamine to negatively charged nucleic acids in fact changes the surface charge of protamine from cationic to anionic [26], [27]. Given the lack of a cationic charge, it is unlikely that the protamine itself is inducing changes itself. Furthermore, we actually looked at foot processes in our studies and showed no changes in the rats that received *shamporter* + control siRNA.

The use of antibodies to direct siRNA to specific target cells or organs has been shown to be a useful approach in other cells [28]. The antibody used in these studies targeted podocytes; however the antigen(s) recognized by the IgG are unknown. While of potential interest, defining these antigens is beyond the scope of this manuscript. Moreover, in its current form, the novelty of effectively delivering siRNA to the glomerulus is itself a huge undertaking and the results are not dampened by the antibody specificity. The data presented here is meant to serve as proof-of-concept that an antibody system can be effective and fairly specific at targeting podocytes. Indeed, this study shows that it is technically feasible, and that others in the future can use this methodology with currently available podocyte specific antibodies. Identifying the antigen(s) and improving specificity of delivery are goals of future studies.

The authors note that parietal epithelial cells (PECs) within the glomerulus have recently been shown to likely serve as progenitors to podocytes and share some common antigens as do the parietal podocyte lining parts of Bowman's basement membrane [29]–[32]. Indeed, we have a manuscript in press adding to this literature [33]. These recent studies have dramatically changed previous notions of “podocyte specificity” using current standard techniques. To this end, the authors believe that no antibody is therefore completely podocyte specific as long as PECs and podocyte gene expression overlaps.

This observation therefore also opens the possibility that PECs serving as progenitors may also be targeted by our delivery system. Thus, care needs to be taken when designing the targets with this system, so as not to interfere with the natural repair system these progenitors may provide in replacing damaged or lost podocytes. Likewise, while we did test for binding of the antibody in several organs as discussed in the manuscript, there is the possibility that the unidentified antigen(s) may be present within other organs and this could lead to off target effects. This is true of many antibody delivery systems.

While we have only demonstrated a transient knockdown of the targeted genes, we believe that repeated injections of *shamporter* may enable longer-term knockdown and studies are ongoing to test this.

One might ask why the delivery of the modified antibody does not induce disease. Studies have shown that a monovalent antibody is not complement activating, which is one of the reasons for the lack of host response and disease induction [16]. Our results were consistent with this observation. Furthermore, in a recently published collaborative study utilizing this antibody, it was shown DAF-deficient T cells are required for induction of disease with the intact version of this antibody [34].

While gene knock-down in animals are typically performed at the genomic level by introducing mutations. These mutations lead to either a permanent or conditional mutant phenotype. In the conditional variant, the phenotype will occur only in a temporary or spatial manner, for example only during certain stages of development or only in the kidney [35], [36]. While these gene knock-downs are valuable and helpful in our understanding of the function of specific genes, there are problems associated with these models. Mutations in essential genes can result in lethality or a renal phenotype that is non-functioning. Additionally, while inducible systems are being developed [37], traditionally mutations can not be switched on and off and therefore the influence of specific genes on the progression of ongoing disease can only be studied within limits. Likewise there is substantial cost in developing these models. A study by the NIH found that generating a transgenic mouse typically takes about one year and costs between one and two hundred thousand dollars [38].

In conclusion, we show that use of a modified antibody to deliver siRNA to podocytes is an effective methodology *in vivo*, without systemic effects.

## Methods

### Immortalized Mouse Podocyte Culture

Immortalized mouse podocytes in culture were utilized as both the antigen to produce an antibody and to test the utility of our siRNA delivery system *in vitro*. Immortalized podocytes were derived from H-2Kb-tsA58 transgenic mice kidneys (ImmortoMouse®; The Jackson Laboratory, Bar Harbor, ME, USA) as recently described [39], [40] ImmortoMouse® harbors a temperature-sensitive SV40 large T cell antigen (tsA58 Tag) under the control of an interferon-γ inducible H-2Kb promoter. Under growth permissive conditions (33°C with 50 units/ml mouse INF-γ) podocytes proliferate and under growth restrictive conditions (37°C in the absence of mouse INF-γ) podocytes stop proliferating and differentiate, displaying many features of podocytes *in vivo*. Podocytes were grown in standard RPMI 1640 medium that contained 10% FBS (Fetalplex™, Gemini Bio-Products, West Sacramento, CA, USA), penicillin (100 U/ml), streptomycin (100 µg/ml), sodium pyruvate (1 mmol/L; (all Irvine Scientific, Santa Ana, CA, USA), HEPES buffer (10 mmol/L; Sigma Chemical Co., St. Louis, MO, USA), and sodium bicarbonate (0.075%; Sigma Chemical Co.). Podocytes were characterized as previously described [41]. Fully differentiated podocytes between 12–18 passages were utilized for our studies.

### Generation of a Podocyte Specific Antibody

In order to devise a novel podocyte specific siRNA delivery system, we developed an antibody that would specifically bind to podocytes. Accordingly, fully differentiated immortalized podocytes (3–5×10^6^ cells) were combined with complete Freund's adjuvant (2 ml) for the first immunization of a sheep. Incomplete Freund's adjuvant was used for subsequent immunizations and administered to the sheep subcutaneously at multiple sites. Following more than 10 cycles of immunizations, the sheep underwent plasmapharesis at peak antibody titer (10 days following immunization). IgG was then isolated from the plasma using caprylic acid followed by ammonium persulfate precipitation [42]. Isolated IgG was dialyzed against PBS and sterile filtered. We and others have previously reported that the injection of a similar anti-podocyte antibody into mice induces experimental glomerular disease [43], [44]. Thus in order to take full advantage of the affinity of the antibody for the podocyte, while at the same time preventing the antibody from inducing disease, the anti mouse podocyte was modified extensively as described below.

### Characterization of a Podocyte Specific Antibody

Western blot analysis was performed on membrane preparations extracted from fully differentiated immortalized mouse podocytes [39], [40] mouse mesangial cells [11], mouse fibroblasts [12], rat proximal tubular epithelial cells [13], and rat glomerular endothelial cells [14], as described below. Protein concentrations were determined using the BCA protein assay (Pierce, Rockford, IL, USA).

### Uptake of Podocyte Specific Antibody by Podocytes

Fully differentiated immortalized mouse podocytes were incubated with 2 mg/ml podocyte specific IgG on ice for 30 minutes to allow binding. Another group of podocytes were incubated with podocyte specific antibody as described, then subjected to additional 30 minute incubation at 37°C to allow internalization of podocyte specific IgG. Cells were lysed in 10 mM Tris buffer (pH 7.5) containing 300 mM sucrose, 1 mM EDTA (all from Sigma-Aldrich, St. Louis, MO), protease inhibitors (Roche, Indianapolis, IN), 50 mM NaF (Sigma-Aldrich), 1 mM Na-orthovanadate (Sigma-Aldrich). The cell homogenate was centrifuged for 2 min at 800×g to clear the lysates from cellular debris and unlysed cells. The supernatant was further centrifuged for 2 min at 2,000×g. The pellet contained the nuclear fraction. The supernatant was further centrifuged for 20 min at 10,000×g to separate cellular organelles from cytosolic proteins. The supernatant was centrifuged for 1 h at 100,000×g resulting in the cytosolic fraction (supernatant). The pellet was washed and centrifuged an additional 1 h at 100,000×g resulting in the membrane fraction (pellet). Membrane and cytosolic subcellular fractions from both groups were analyzed by western blot analysis for sheep IgG heavy chain. Alpha/beta tubulin cytoplasmic) and sodium potassium adenosine-triphosphatase (membrane) were used as loading controls for each fraction.

### Modification of Sheep Anti-Mouse Podocyte Antibody

Purified IgG (10 mg) was selectively cleaved at the internal heavy-chain disulfide bond with 2-MEA at a concentration of 50 mM (2-mercaptoethylamine-HCl; Pierce, Rockford, IL, USA) (**Figure 1, step 1**). 2-MEA was then removed using a desalting column. Cleaving the polyvalent antibody with 2-MEA produced two monovalent antibody fragments, thereby destroying the ability of the antibody to activate complement [15], [16], while leaving the antigen recognition sites intact.

Following IgG cleavage, the sulfohydryl group was utilized to link a defined number of Neutravidin molecules (n = 1) to each monovalent IgG. NeutrAvidinTM protein was conjugated to the monovalent IgG using EZ-Link kit per the manufacturer's instructions (EZ-Link® Maleimide Activated NeutraAvidinTM Protein; Pierce, Rockford, IL, USA). (**Figure 1, step 2**)

Next, we added protamine, a universal adaptor for siRNA and other nucleic acids onto the monovalent antibody [26]. Protamine is a polycationic nuclear protein (MW ∼4000 Da) that binds DNA during the haplophase in the absence of histones [45]. Protamine stabilizes nucleic acids and also prevents siRNA from enzymatic degradation, thereby increasing the siRNA half life [46]. Protamine sulphate is an FDA approved compound and its safety is well documented, making it suitable even for applications in human therapy [47]. Protamine sulphate (Salmine P4020, Sigma, St. Louis, MO, USA) was biotinylated via it's N-terminal amino group using EZ-Link® Sulfo-NHS-Biotinylation Kit per the manufacturer's instructions (Pierce, Rockford, IL, USA) (**Figure 1, step 3**). The modified antibody containing the Neutravidin site and the biotinylated protamine were mixed in a ratio of 1∶1.2 and incubated for 60 minutes on a rotor at 4°C. To remove unbound protamine and conjugation buffer, the antibody-protamine complex was dialyzed overnight against phosphate buffered saline pH 8.3 at 4°C in a 10,000 molecular weight cut-off Slide-A-Lyzer® cassette (Pierce, Rockford, IL, USA) (**Figure 1, step 3**). Because protamine is positively charged, it attracts the negative charge of siRNA and allows transport of siRNA directly to the podocyte (**Figure 1, steps 4 & 5**). We called this novel podocyte specific siRNA delivery system *shamporter* (**sh**eep **a**nti **m**ouse **po**docyte trans**porter**).

### Design and Synthesis of siRNA

In order to test the feasibility of the delivery system, we employed siRNA to genes that are constitutively expressed in podocytes. Accordingly, the following commercial siRNAs were utilized: (i) siRNA directed against the cyclin dependent kinase inhibitor p57Kip2 (M-062494, Dharmacon Inc., Chicago, IL, USA), (ii) siRNA directed against glyceraldehyde 3-phosphate dehydrogenase (GAPDH) and (iii) siRNA directed against cyclin dependent kinase five (CDK5) 4390849 & 60726 Applied Biosystems, Austin, TX, USA) and (iv) Silencer Negative Control #1 siRNA (AM4635 Applied Biosystems, Austin, TX, USA) was utilized as a negative control. (v) Additionally, siRNA directed against nephrin (Npsh1) and TRPC6 were designed using the RiDDLE database [48] and synthesized using SilencerTM siRNA Cocktail Kit, (Ambion, Applied Biosystems, Austin, TX, USA) according to the manufacturer's protocol.

### *Shamporter In Vivo*

To ensure that there was no non-specific binding of the anti-podocyte antibody in organs outside the kidney, male Balb/c mice (n = 4, bodyweight 22–35 g) were injected with a single dose (15 mg/20 g BW) of mouse anti-podocyte antibody into the tail vein. Control animals (n = 4) received normal sheep IgG (15 mg/20 g BW). After 72 hours, mice were sacrificed and biopsies were taken from kidney, heart, brain, lung, spleen, liver, colon and muscle, snap frozen and embedded in OCT for immunostaining for sheep IgG. Immunofluorescent staining for sheep IgG was performed on 4 µm frozen sections from each organ to determine if podocyte antibody deposited in these organs. Sections were fixed in methanol for 30 minutes at −20°C, washed in phosphate buffered saline, and incubated with fluorescein conjugated rabbit anti-sheep IgG antibody (Abcam, Cambridge, MA, USA).

### Delivering siRNA to Podocytes *In Vivo*

Nephrin has been well studied and mutations or alterations of the subcellular level of nephrin lead to proteinuria [16], [21]. Thus, in order to test the delivery of *shamporter* to podocytes *in vivo*, siRNA directed against nephrin was linked to *shamporter* and used as follows. Six normal adult male Sprague-Dawley rats (Charles River Labs, Boston, MA, USA) (144 g±10) were uninephrectomized to reduce the glomeruli number, then injected intravenously with 0.3 µmol of modified anti podocyte antibody (*shamporter*) in combination with 2 µmol siRNA directed against nephrin. Control animals received 0.3 µmol *shamporter* in combination with 2 µmol non-functional control siRNA. Normal sheep IgG in 500 µl sterile PBS was used as vehicle for *shamporter* and siRNA to control non-specific IgG binding.

To silence TRPC6 expression in podocytes *in vivo*, we injected *shamporter* + TRPC6 siRNA in four male Sprague-Dawley rats (150–190 g). Again, animals were uninephrectomized to reduce the glomeruli number. Nephrectomized kidneys served as normal control kidneys. After five days each rat was injected intravenously with 2 mg *Shamporter* + 2 mg normal sheep IgG + 50 nM TRPC6 siRNA (2 rats) or 50 nM negative control siRNA (2 rats). The concentration of siRNA was calculated on an estimated blood volume of 10 ml. Experimental and control animals were sacrificed after 48 hours, kidney biopsies were harvested and processed for further analysis. Glomerular preparations were performed, as described before [49], to determine levels of TPRC6 in podocytes by western blot analysis.

All interventions were performed under a general anesthetic composed of 50% ketamine, 25% xylazine, 15% azepromazine (all Phoenix Pharmaceuticals, St. Joseph, MO, USA) and 10% Ringer's solution (Baxter Healthcare, Deerfield, IL) at a dose of 100 µl per 100 g body weight. Animals were housed with free access to food and water. Experimental and control animals were sacrificed after 72 hours, and kidney biopsies were obtained, fixed and immunostained as described below.

### Measuring Renal Function

In order to assess renal function, urine was collected by placing animals in metabolic cages for 12 hours, during which time water was supplied without restriction. Urine was collected before *shamporter* injection (baseline), and 24, 48, 72, 96 hours, and 7 days after *shamporter* injection. Total protein excretion was determined by the sulfosalicylic acid turbidity method as previously described [51]. Urine creatinine excretion was measured using a commercially available colorimetric microplate assay based on the Jaffe reaction (Colorimetric Creatinine Assay Kit, CR01, Oxford Biomedical Research, MI, USA) [52].

### Immunohistochemical Staining

To ensure that the levels of nephrin were reduced, indirect immunoperoxidase staining was performed for nephrin and podocin on serial sections from formalin fixed paraffin embedded kidney tissue as previously described [50], [53].

Briefly, paraffin was removed with Histoclear (National Diagnostics, Atlanta, GA, USA) and sections were re-hydrated in ethanol. Antigen retrieval was performed by boiling sections in 1 mM ethylenediaminetetraacetic acid (EDTA) buffer pH 6.0. Endogenous peroxides activity was quenched with 3% hydrogen peroxide and non-specific protein binding was blocked with Background Buster (Accurate Chemical&Scientific Corporation, Westbury, NY, USA). Endogenous biotin activity was quenched with Avidin/biotin blocking kit (Vector Laboratories, Burlingame, CA, USA). After blocking sections were incubated with either polyclonal nephrin or podocin antibodies (antibodies listed below) overnight at 4°C, unbound antibody was removed by washing in PBS and biotin-labelled goat antibody directed against guinea pig or rabbit IgG was applied for 1 hour at room temperature.

R.T.U. Vectastatin kit was applied (Vector Laboratories) and staining was visualized by precipitation of Diaminobenzidine (DAB) (Sigma Aldrich) for nephrin or Vector SG substrate kit as per manufacturer's instructions for podocin. The primary antibodies were omitted as negative controls.

### Immunofluorescent Staining

Frozen 4 µm sections were fixed in 100% methanol and incubated with rabbit TRPC6 antibody followed by FITC labelled mouse anti-rabbit IgG, or biotin labelled monoclonal anti rat c5b-9 followed by streptavidin-fluorescein or FITC labelled goat polyclonal to Rat C3.

### Western Blot Analysis

Western blot analysis was also performed as described previously to further quantitate any decrease in target gene protein levels [48]. Briefly, 20 µg protein extracts in Laemmli buffer containing 5% 2-Mercaptoethanol were separated by sodium dodecyl sulfate–polyacrylamide (SDS) gel electrophoresis and transferred to polyvinylidene difluoride membrane. After blocking non-specific binding with 5% non fat dry milk or 5% bovine serum albumin (Sigma Chemicals, St. Louis, MO, USA), membranes were incubated overnight at 4°C with primary antibodies (see below). Secondary anti-rabbit IgG horseradish peroxidase antibody (GE Healthcare, Piscataway, NJ, USA) was applied for 1 hour at room temperature followed by enhanced chemoluminescence (ECL) western blotting system (GE Healthcare, Piscataway, NJ, USA).

### Antibodies

The following antibodies were utilized: Guinea pig polyclonal to Nephrin, RDI-PROGPN2 (Fitzgerald, Concord, MA, USA); Anti human Podocin IgG, PODO11-A (Alpha Diagnostic, San Antonio, TX, USA); Rabbit polyclonal to TRPC6, ACC-017 (Alomone labs, Jerusalem, Israel); Rabbit polyclonal to TRPC6, ab47679 (Abcam, Cambridge, MA, USA); FITC labelled Goat polyclonal to Rat C3 (MP Biochemicals, Solon, Ohio, USA); Biotin labelled monoclonal anti rat c5b-9 (SantaCruz Biotechnology, Inc., Santa Cruz, CA. USA); Streptavidin-fluorescein & Donkey ECL anti rabbit IgG (HRP), NA934 (GE Healthcare, Piscataway, NJ, USA); Rabbit polyclonal anti-guinea pig IgG H&L (HRP), ab6771 (abcam, Cambridge, MA, USA); FITC labelled rabbit polyclonal anti sheep IgG H&L, ab6743 (abcam, Cambridge, MA, USA); FITC labelled mouse anti-rabbit IgG (Jackson, West Grove, PA, USA); Biotin labelled goat polyclonal anti-guinea pig IgG, BA-7000 and biotin labelled goat polyclonal anti rabbit IgG (both Vector Laboratories, Burlingame, CA, USA).

### Morphometric Evaluation

To determine the average foot process width, 20 electron microscopy images from 4 animals of each group, at 7100× magnification were examined. On each image, the curved total length of the basement membrane (BML) was measured using Image J [54]. Counting the number slit diaphragms; the average foot process width (Wp) was calculated using the formula below [55].
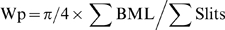



### Statistical Analysis

Results are expressed as mean ± standard error of the mean. Paired *t*-test was used in the densitometry analysis. Statistical significance was defined as *p*<0.05; test was performed using Microsoft Excel. ANOVA analysis (p<0.05) was performed to calculate intra group differences of the *in vivo* experiment [56]. Protein/creatinine ratios are expressed as median ± max and min. Student *t*-test was used to compare protein/creatinine ratios of treated to untreated rats, statistical significance was defined as *p*<0.05.

### Animal Experiments

Animals used in this study were housed in the University of Washington animal facility according to standardized specific pathogen-free conditions. Experimental procedures involving animals were reviewed and approved by the Animal Care Committee of the University of Washington. Researchers handling animals were trained and certified according the guidelines of the American Association for Laboratory Animals (ALAAS).

## Supporting Information

Figure S1 *Immunofluorescent staining of anti mouse podocyte antibody in different organs following tail vein injection*. (A) Sheep anti mouse podocyte antibody staining was detected only in the glomerulus, and this was in a podocyte distribution (B). Staining was absent in the control animals injected with normal sheep IgG. (C) In the spleen, there was staining for both anti-mouse podocyte antibody and normal sheep IgG injected control (D) consistent with IgG trapping. (E) Antibody staining was detected in brain vasculature. (F) Staining for normal sheep IgG was absent in the brain.
(1.47 MB TIF)

Figure S2 *Complement staining in shamporter injected animals*. Immune fluorescence staining for showing absence of complement activation in shamporter injected rats. Animals injected with shamporter + control siRNA or shamporter +nephrin siRNA do not show positive signals for complement factors C5b-9 (B+C) or C3 (E+F). Rat with Passive Heymann Nephritis was used as a positive control shows typical staining for C5b-9 and C3 (A+C).
(1.28 MB TIF)
