# Supplementary figures and images for: Novel siRNA Delivery System to Target Podocytes In Vivo
Source: PLoS One. 2010 Mar 1;5(3):e9463. doi: 10.1371/journal.pone.0009463 (PMC2830889; doi:10.1371/journal.pone.0009463)

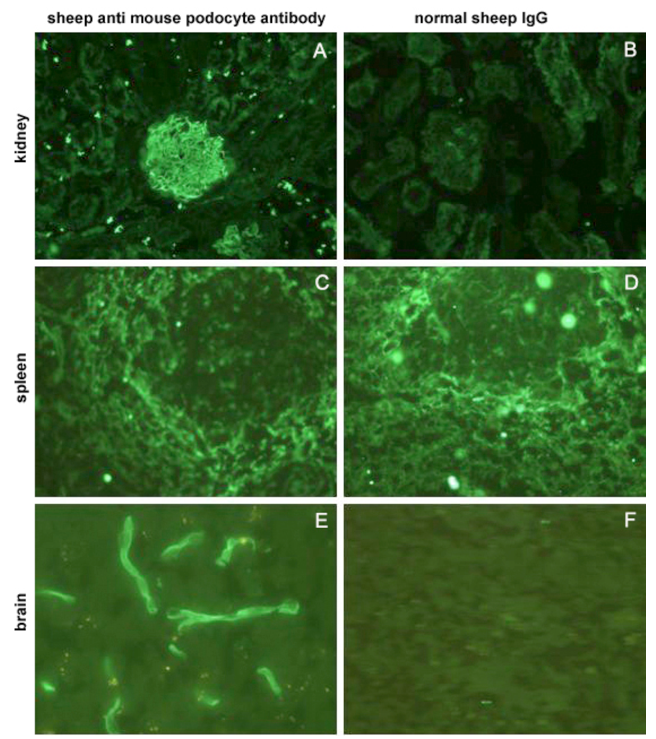

Supplement: Figure S1 — Immunofluorescent staining of anti mouse podocyte antibody in different organs following tail vein injection. (A) Sheep anti mouse podocyte antibody staining was detected only in the glomerulus, and this was in a podocyte distribution (B). Staining was absent in the control animals injected with normal sheep IgG. (C) In the spleen, there was staining for both anti-mouse podocyte antibody and normal sheep IgG injected control (D) consistent with IgG trapping. (E) Antibody staining was detected in brain vasculature. (F) Staining for normal sheep IgG was absent in the brain. (1.47 MB TIF) [file pone.0009463.s001.tif]

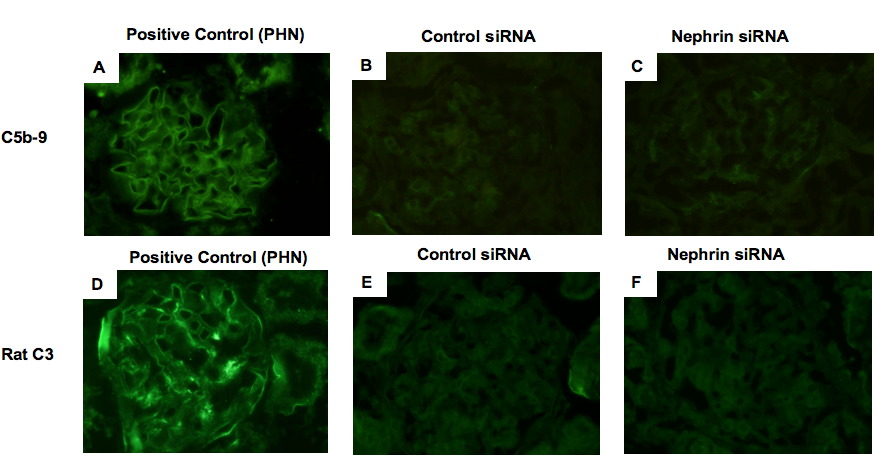

Supplement: Figure S2 — Complement staining in shamporter injected animals. Immune fluorescence staining for showing absence of complement activation in shamporter injected rats. Animals injected with shamporter + control siRNA or shamporter +nephrin siRNA do not show positive signals for complement factors C5b-9 (B+C) or C3 (E+F). Rat with Passive Heymann Nephritis was used as a positive control shows typical staining for C5b-9 and C3 (A+C). (1.28 MB TIF) [file pone.0009463.s002.tif]
